# Application of an Arbitrary Lagrangian–Eulerian Method to Modelling the Machining of Rigid Polyurethane Foam

**DOI:** 10.3390/ma14071654

**Published:** 2021-03-28

**Authors:** Zdenek Horak, Petr Tichy, Karel Dvorak, Miloslav Vilimek

**Affiliations:** 1 Department of Technical Studies, College of Polytechnics Jihlava, Tolsteho 16, 58601 Jihlava, Czech Republic; karel.dvorak@vspj.cz; 2 Department of Mechanics, Biomechanics and Mechatronics, Faculty of Mechanical Engineering, Czech Technical University in Prague, Technicka 4, 16607 Prague, Czech Republic; petr.tichy@fs.cvut.cz (P.T.); miloslav.vilimek@fs.cvut.cz (M.V.)

**Keywords:** PUR foam, machining, finite element (FE) analyses, Arbitrary Lagrangian Eulerian

## Abstract

Rigid polyurethane (PUR) foam, which has an extensive range of construction, engineering, and healthcare applications, is commonly used in technical practice. PUR foam is a brittle material, and its mechanical material properties are strongly dependent on temperature and strain rate. Our work aimed to create a robust FE model enabling the simulation of PUR foam machining and verify the results of FE simulations using the experiments’ results. We created a complex FE model using the Arbitrary Lagrangian–Eulerian (ALE) method. In the developed FE model, a constitutive material model was used in which the dependence of the strain rate, damage initiation, damage propagation, and plastic deformation on temperature was implemented. To verify the FE analyses’ results with experimentally measured data, we measured the maximum temperature during PUR foam drilling with different densities (10, 25, and 40 PCF) and at various cutting speeds. The FE models with a constant cutting speed of 500 mm/s and various PUR foam densities led to slightly higher T${}_{max}$ values, where the differences were 13.1% (10 PCF), 7.0% (25 PCF), and 10.0% (40 PCF). The same situation was observed for the simulation results related to various cutting speeds at a constant PUR foam density of 40 PCF, where the differences were 25.3% (133 mm/s), 10.1% (500 mm/s), and 15.5% (833 mm/s). The presented results show that the ALE method provides a good match with the experimental data and can be used for accurate simulation of rigid PUR foam machining.

## 1. Introduction

Rigid polyurethane (PUR) foam has been successfully used in many industries [1,2] since 1937, when Otto Bayer first synthesised it [3]. PUR foam can be used with a wide range of moulds, and its mechanical properties can be varied, making it suitable for use in a wide range of applications. The structural stability and mechanical properties of PUR foam are highly dependent on several physical parameters. The most important factors influencing the mechanical properties of PUR foam are its temperature [4,5], the strain rate [6,7], exposure to UV radiation, and extent of oxidation [8,9]. All of these factors degrade its mechanical properties.

One common application of PUR foam is the development and testing of medical devices [10,11]. For this purpose, certified PUR foam (Sawbones, Vashon, WA, USA) with standardized structure and density are used. Hollensteiner [12] and Oroszlany [13] experimentally evaluated that the mechanical properties (e.g., modulus of elasticity and ultimate strength) of PUR foam are similar to those of bone tissue, thereby making PUR foam suitable for tests of medical devices. PUR foam as standardized material is optimal for testing; however, its thermomechanical properties differ completely from those of bone tissue, and whether PUR foam is ideal for testing instruments used in surgery (e.g., drills and mills) is uncertain.

The phenomenon of heat generation during machining is very complicated because it is influenced by numerous factors, including heat-induced changes in the properties of the machined material. The amount of heat increases primarily with increasing rate of plastic deformation during machining and increasing friction between the machined material and the tool. Arrazola [14] and others authors (e.g., [15,16]) have presented the results of numerical finite element (FE) simulations of machining, where heat generation and its effect on the machined material and the tool itself were modelled. However, all of these works have focused on the analysis of thermally stable metal materials.

To the best of our knowledge, no FE simulations of machining and analysis of heat generation during machining of PUR foam, which is a relatively thermally unstable material, have been reported. Therefore, this work aims to design and validate a suitable method of FE simulations for modeling PUR foam machining. The next aim was to experimentally measure the mechanical properties of PUR foam, defined as a temperature function, and to use these data as inputs for FE simulations. To validate the results realised for FE analyses simulating PUR foam machining, we used the maximum temperature on the tool’s tip during machining. Therefore, the final aim was experimentally measurement of the maximal temperature during drilling into PUR foam for different cutting conditions.

## 2. Materials and Methods

### 2.1. Experimental Measurements of Material Properties of PUR Foam as a Function of Temperature

To carry out the analyses, we first experimentally measured the material properties of rigid PUR foam under both tension and compression at different temperatures so that these values could be used as input parameters for the FE simulations. To ensure similar conditions, experimental measurements and subsequent FE analyses were performed on PUR foam blocks (Sawbones, Vashon Island, WA, USA). Experimental measurements were realised on PUR foam samples with three density values—10, 25, and 40 PCF (Pounds per Cubic Foot) density in SI units 160.32, 400.46, and 640.74 kg/m${}^{3}$. Tensile and compression tests were realised on the testing system ElectroPuls E10000 (Instron, Norwood, MA, USA) with a video extensometer when tests were realised in a controlled environment by the heating chamber. Tensile tests were realised on seven specimens for all analyzed PUR foam densities with specimen dimensions of 10×10×15 mm. Compression tests were realised on five samples from each PUR foam density on samples 10×10×15 mm under the same conditions as the tensile tests. The tested specimens were loaded by force until their destruction in temperature environments of 25, 90, and 155 ${}^{\circ }$C. Both types of tests were load by a strain rate of 45 mm/min.

### 2.2. Experimental Measurement of Temperature on Tool’s Tip during Drilling

**Figure 1:**
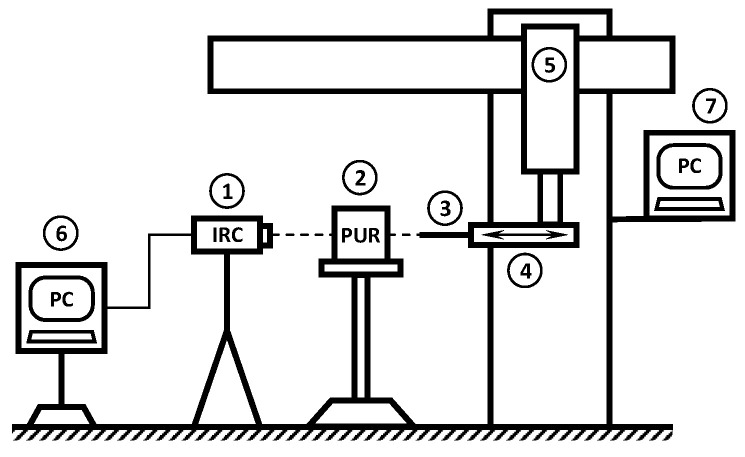
Experimental setup: 1—IR camera with connection to PC; 2—block of PUR foam; 3—drill; 4—dental drilling machine; 5—CNC milling machine with horizontal movement; 6 and 7—PC for drill movement controlling and recording data from IR camera.

One of the planned outputs of the FE analyses was the distribution of the heat on the cutting edge of the tool. For this reason, a series of experimental measurements were carried out to determine the maximum temperature at the tip of the drill when drilling into PUR foams with different densities. Experimental measurements were performed on PUR foam samples with a density of 10, 25, or 40 PCF, where the sample dimensions were 15×15×10 mm. The drilling was performed with a dental drilling machine SI 923 Implantmed (W & H Dentalwerk GmbH, Laufen, Germany) on a 2.9 mm diameter drill BioniQ (LASAK Ltd., Prague, Czech Republic) with a cutting speed of 800, 3000, or 5000 rpm and an axial speed of 10, 30, or 45 mm/min, respectively. Seven samples were measured for each combination of density and cutting speed. The maximum temperature T${}_{max}$ on the drill bit was measured using an IR camera FLIR E40 (FLIR Systems Inc., Wilsonville, OR, USA), at the time the drill passed through the drill sample (see Figure 1). No coolant was used to cool the drill during the experiment.

### 2.3. FE Simulation of the Machining

In this study, FE simulations of PUR foam machining of various densities and cutting speeds were performed. PUR foams are dependent on the crosslinking of the polymer chains [17]. However, in our work, PUR foam was modeled in the performed FE analyses as a material with global material properties defined by stress–strain dependencies. The stress–strain description of the material already includes the influence of the material’s structure and character on a micro or molecular level. The PUR foam specimens were machined using a tool with edge roundness r=0.03 mm, a 10° rake angle, and 10° clearance angles. The chip thickness was constant t=0.2 mm, and the cutting speed vc was 133, 500, and 833 mm/s for all three PUR foam densities, corresponding to the drill speed (800, 3000, and 5000 rpm, respectively) used in the experiments.

PUR foam machining simulations were realised as a 2D task using the ALE formulation in ABAQUS/Explicit. ALE is a method that uses quality mesh throughout an analysis, even in case of large deformations or material damage, when the mesh is deformed independently of the material. Coupled thermal-stress analysis was used to affect temperature, where the output is the distribution of the temperature field in the machined material and cutting tool. During machining, the cutting edge of the tool compresses the workpiece and deforms via elastic and plastic deformation. The work associated with plastic deformation is transformed into heat. In the cutting plane, the chip shifts under high pressure, which is accompanied by considerable friction, and the chip is then rubbed across the face of the tool. Behind the cutting edge, because of the elastic component of the deformation, the material is pushed towards the back surface, and friction occurs again. Friction always results in the conversion of mechanical energy into heat. The heat is conducted to the chip, tool, and workpiece and is radiated to the surroundings. In the ALE model, heat transfer is only allowed on surfaces where contact occurred between the tool and the PUR foam.

**Figure 2:**
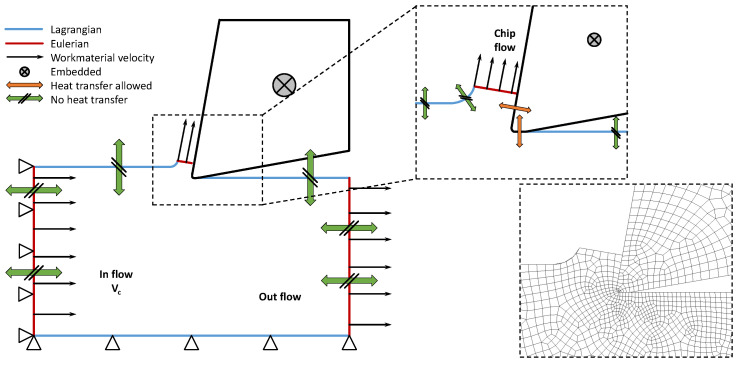
Description of the Arbitrary Lagrangian–Eulerian model and detail of the mesh.

In our study, the inflow and outflow surfaces of the model corresponded to Eulerian surfaces, including two outflow surfaces: the vertical surface of the workpiece and the surface on the top of the chip. The remaining surfaces were defined as Lagrangian surfaces. On the inflow surface, the node displacement was constrained in the horizontal direction, and the velocity of the material was equal to the cutting speed $v_{c}$. Bottom Lagrangian surfaces were constrained to be fixed in the vertical direction. The cutting tool was defined as the rigid body where a reference point was embedded (see Figure 2). Heat transfer was not allowed in any of the remaining areas (see Figure 2). The contact between the tool and the material was modelled as normal, which enabled separation after contact with a constant friction coefficient f=0.15. Conductive heat transfer, defined by conductance k [W/(m·K)] as a function of closure c, was allowed. In actual FE analyses, the linear function of conductance k=0.01 for c=0 and k=0 for c=10 was used. The friction of the contact surfaces dissipates the energy that was completely converted into heat in our model when the heat was distributed equally between the two surfaces. An initial condition, temperature $T_{0}=293$ K, was applied throughout the model. In all of the realised fully coupled thermal-stress analyses, we specified an inelastic heat fraction to provide for inelastic energy dissipation as a heat source. Inelastic heat fractions were used for large inelastic strain simulations, where the material deformation depends on the material properties influenced by heating. The value of inelastic heat fractions was set to 0.8 in all of the simulations.

In the FE simulations, four-node bilinear plane strain thermally coupled quadrilateral elements with bilinear displacement and temperature, reduced integration were used. The global element size was 0.2 mm when contact surfaces were refined to the element size of 0.01 mm. Rigid PUR foam was modelled as a thermoelasto-viscoplastic material [18]. The elastic region of the material model was defined as temperature-dependent when the material properties were experimentally measured (see Section 3.1).

The selected material parameters are listed in Table 1. The Johnson–Cook material model was utilised; its flow stress is dependent on strain, strain rate, and temperature [19,20,21]. In Johnson–Cook hardening (adapted from [22]), the static yield stress is defined as

(1) $$\begin{array}{c} \bar{\sigma }=\left[A+B{\left({\bar{\varepsilon }}^{pl}\right)}^{n}\right]\left[1+C\;ln\left(\frac{{\dot{\bar{\varepsilon }}}^{pl}}{{\dot{\varepsilon }}_{0}}\right)\right]\left[1-\Theta^{m}\right] \end{array}$$

where *A* is the yield strength, *B* is the hardening modulus, *C* is the coefficient of strain rate sensitivity, *n* is the hardening coefficient, *m* is the thermal softening coefficient, $\Theta =\left(T-T_{0}\right)/\left(T_{m}-T_{0}\right)$ where $T_{0}$ is the transition temperature and $T_{m}$ is the melting temperature, ${\bar{\varepsilon }}^{pl}$ is the Mises equivalent plastic strain, ${\dot{\bar{\varepsilon }}}^{pl}$ is the Mises equivalent plastic strain rate, and ${\dot{\varepsilon }}_{0}$ is the reference strain rate [23,24]. Used material constants were obtained from experimental measurements on PUR foam. (see Table 2).

The fracture model used for ductile materials consists of two phases: a damage-initiation phase and a damage-evolution phase. The primary mechanism that causes the ductile fracture of a ductile material is the nucleation, growth, and coalescence of voids. The ductile criterion is a model for predicting material damage when equivalent plastic strain is defined as a function strain rate and stress triaxiality

(2) $$\begin{array}{c} {\bar{\varepsilon }}_{D}^{pl}\left(\eta ,{\dot{\varepsilon }}^{pl}\right) \end{array}$$

where η=−p/q is the stress triaxiality, *p* is the pressure stress, and *q* is the Mises equivalent stress (adapted from [22]). The damage initiation criterion was met when the following was applied:

(3) $$\begin{array}{c} \omega_{D}=\int \frac{d{\bar{\varepsilon }}^{pl}}{{\bar{\varepsilon }}_{D}^{pl}\left(\eta ,{\dot{\varepsilon }}^{pl}\right)}=1 \end{array}$$

where $\omega_{D}$ is a state variable that increases together with plastic deformation (adapted from [22]). The incremental increase of $\omega_{D}$ is for each increment determined according to

(4) $$\begin{array}{c} \Delta \omega_{D}=\frac{\Delta {\bar{\varepsilon }}^{pl}}{{\bar{\varepsilon }}_{D}^{pl}\left(\eta ,{\dot{\varepsilon }}^{pl}\right)}\geq 0. \end{array}$$

**Figure 3:**
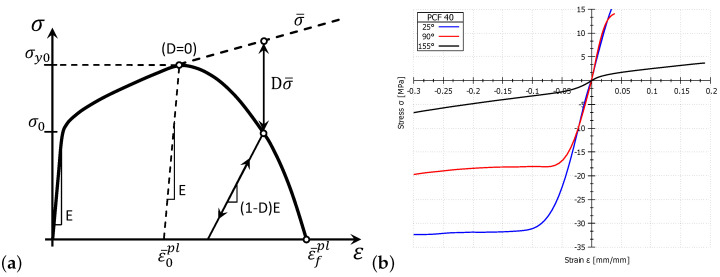
(**a**) Typical stress–strain curve for a material model with failure due to progressive damage simulated in Abaqus for specimen during a simple tensile test [22], (**b**) typical stress–strain curves for sample loaded by a 45 mm/min strain rate (40 PCF PUR foam).

The value of equivalent plastic strain as a function of temperature for all PUR foams is given in Table 1. After the criteria for damage initiation have been established, the damage evolution must be set. For the damage evolution in ductile material, we modeled the stiffness decreasing until failure. Figure 3 presents a typical stress–strain curve for a material model with isotropic hardening until failure due to progressive specimen damage during a simple tensile test. The solid curve presented material model with damage and dashed curve material without damage.

In the graph in Figure 3, the yield stress $\sigma_{0}$, the equivalent plastic strain ${\bar{\varepsilon }}_{0}^{pl}$ and the equivalent plastic strain at failure ${\bar{\varepsilon }}_{f}^{pl}$. Upon reaching the damage initiation criterion, the damage variable increases by (adapted from [22])

(5) $$\begin{array}{c} \dot{d}=\frac{{\dot{u}}^{pl}}{{\bar{u}}_{f}^{pl}} \end{array}$$

where ${\bar{u}}_{f}^{pl}$ is the equivalent plastic displacement at failure, defined by

(6) $$\begin{array}{c} {\bar{u}}_{f}^{pl}=\frac{2G_{f}}{\sigma_{y0}} \end{array}$$

where $\sigma_{y0}$ is the yield stress when the failure criterion is reached and G${}_{f}$ is the fracture energy.

## 3. Results

### 3.1. Material Properties of PUR Foam as a Function of Temperature

Experimental measurements were conducted to determine the temperature dependence of the mechanical properties of the PUR foams. The mean tensile and compressive modulus of the tested PUR foams with various densities, together with the mean size of tensile and compressive strength, were determined from experimental measurements. The typical stress–strain curve of the 40 PCF PUR foam is presented in Figure 3b for all three analysed temperatures (25, 90, and 155 Â ${}^{\circ }$C). Results of the realised experimental measurement are presented in Table 3.

### 3.2. Experimental Measurement of Heat on the Drill during Drilling

Experiments were conducted to determine the maximum temperature at the drill tip when drilling PUR foams of different densities at three cutting speeds (800, 3000, and 5000 rpm). Seven test samples were measured for each combination of density and cutting speed. The maximum temperature at the tip of the drill bit was measured using an infrared (IR) camera with a recording frequency of 10 Hz. The average value of the maximum temperature was obtained from the measured values. The results of the experimental measurements are summarised in Table 4. The results of experimental measurements of maximum temperature T${}_{max}$ show that the temperature at the drill bit increases with increasing density of the PUR foams and increasing cutting speed. Interestingly, the increase in T${}_{max}$ between the 10 PCF and 25 PCF materials is substantial: 74.3% for 800 rpm, 57.0% for 3000 rpm, and 67.1% for 5000 rpm. By contrast, the increase in T${}_{max}$ between the 25 PCF and 40 PCF materials was relatively small (32.3% for 800 rpm, 20.0% for 3000 rpm, and 16.9% for 5000 rpm).

### 3.3. FE Simulation of the Machining

**Figure 4:**
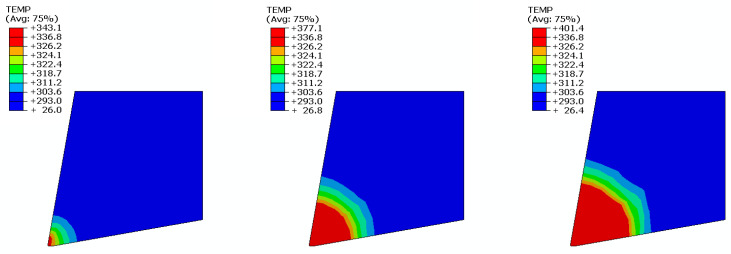
Temperature T [K] distribution on the tool’s tip for constant cutting speed 500 mm/s during machining materials with different densities: 10 PCF (**left**), 25 PCF (**middle**), and 40 PCF (**right**).

**Figure 5:**
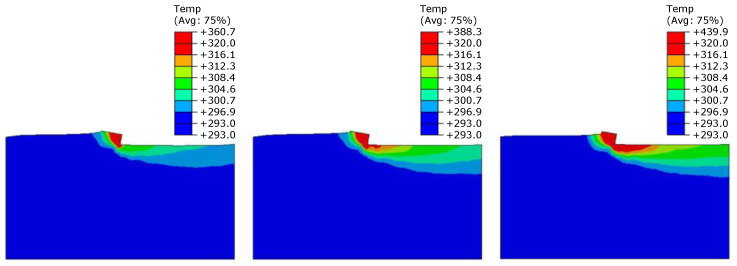
Temperature T [K] distribution during machining of the PUR foam with density 40 PCF : cutting speed 133 mm/s (**left**), 500 mm/s (**middle**), and 833 mm/s (**right**).

The outputs of the FE analyses were the T${}_{max}$ at the tool cutting edge and the distribution of heat field in the rigid PUR foams with different densities (10, 25, and 40 PCF) during machining at various cutting speeds (133, 500, and 833 mm/s). The first parameter evaluated was the T${}_{max}$ at the tip of the cutting tool, which can be compared with experimentally measured values. The T${}_{max}$ values obtained from the FE analyses are given in Table 5 and Figure 4; the measured T${}_{max}$ values are reported in Table 4. The FE simulation results are in line with the expectation that with increasing temperature and cutting speed, the tool’s tip and PUR foam temperature increase. Temperature distribution during machining of the PUR foam 40 PCF for various cutting speeds is presented in Figure 5.

**Figure 6:**
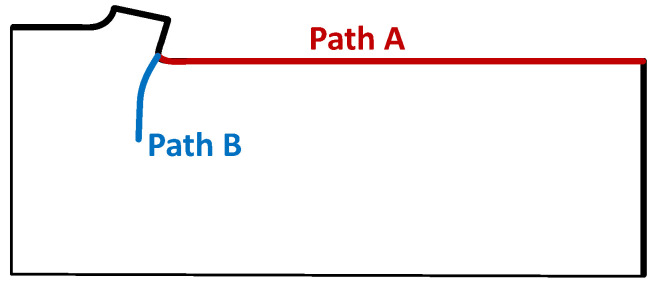
Schematic of Paths A and B on the model, where the temperature was analysed.

The second parameter evaluated was the temperature along two paths on the model. Path A started from the point of material contact with the cutting edge and proceeded towards the rear of the specimen. Path B started at the same position as Path A but was directed towards the centre of the rigid PUR foam (Figure 6).

**Figure 7:**
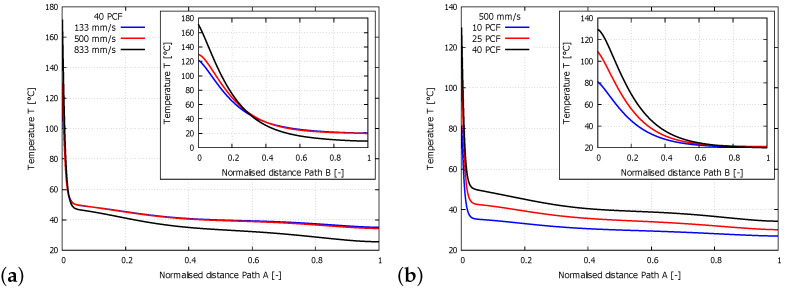
(**a**) Graph of the magnitude of the temperatures on Paths A and B for a constant density (40 PCF) with different cutting speeds. (**b**) Graph of the magnitude of the temperatures on Paths A and B for a constant cutting speed (500 mm/s) with different sample densities.

The values of the temperatures on both paths for evaluating the effect of the cutting speed on the model with a constant sample density (40 PCF) are shown in the graph in Figure 7a. On Path A, the temperature range for the cutting speed of 133 mm/s was in the range 121.8–35.0 ${}^{\circ }$C, that for the speed of 500 mm/s was in the range 129.8–34.3 ${}^{\circ }$C, and that for the speed of 833 mm/s was in the range 171.5–25.5 ${}^{\circ }$C. On Path B, the temperature for the cutting speed of 133 mm/s was in the range 121.8–20.3 ${}^{\circ }$C, that for the speed of 500 mm/s was in the range 129.8–20.0 ${}^{\circ }$C, and that for the speed of 833 mm/s was in the range 171.5–9.2 ${}^{\circ }$C. The temperatures on both paths for evaluating the effect of density on the model with a constant cutting speed (500 mm/s) are shown in Figure 7b. On Path A, the temperature for the 10 PCF sample was in the range 81.4–26.9 ${}^{\circ }$C, that for the 25 PCF sample was in the range 109.4–30.1 ${}^{\circ }$C, and that for the 40 PCF sample was in the range 129.8–34.3 ${}^{\circ }$C. On Path B, the temperature for the 10 PCF sample was in the range 81.4–20.4 ${}^{\circ }$C, that for the 25 PCF sample was in the range 109.4–21.0 ${}^{\circ }$C, and that for the 40 PCF sample was in the range 129.8–20.0 ${}^{\circ }$C.

## 4. Discussion

The process of machining PUR foam was simulated by means of FEM as orthogonal cutting. Validation of the results from FE simulations was realised using experimentally measured data obtained from drilling into PUR foam. Although these types of machining are different, data can be compared with each other. In FE simulations, the orthogonal model’s cutting speed was the same as the real drilling speed. Likewise, the cutting tool’s dimensions (contact area) in the FE simulation were identical to those in the experimental drilling measurements. The used ALE method does not carry out the physical removal of material or generate chips during machining. Therefore the difference between the chip formation process during orthogonal machining and drilling is not relevant. The analysis’s main goal was to determine the temperature at the tip of the tool, where heat generation depends on the cutting speed, the shape of the tool, and the physical conditions for heat conduction between the material and the tool. All the above parameters were the same for FE simulations and experimental measurements, and therefore, in our opinion, it is possible to compare the results of FE simulations and experimental measurements.

One factor that influenced the FE simulation results was the strain rate. Experimental measurements of PUR foam material parameters were performed at a cutting speed of 45 mm/min; however, in the FE simulations, the cutting speed ranged from 133 to 833 mm/s. During machining, the material is deformed at a velocity substantially greater than the sample loading speed in the experimental measurements. The mechanical properties of the PUR foam are highly dependent on the rate of deformation [7]; thus, this effect cannot be neglected. More accurate (depending on the temperature and strain rate) material parameters than those we have measured experimentally have not been published elsewhere. Therefore, using more appropriate material data as input data for FE simulations was not feasible.

**Figure 8:**
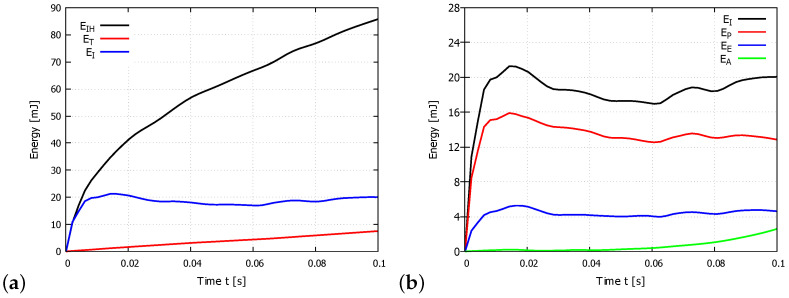
Graphs of energies in the FE model during cutting simulation (density 40 PCF): (**a**) internal heat energy $E_{IHE}$, total energy $E_{T}$, and total strain energy $E_{EI}$; (**b**) total strain energy $E_{I}$, inelastic dissipated energy $E_{P}$, elastic strain energy $E_{E}$, and artificial strain energy $E_{A}$.

The main aim of this work was to carry out a numerical FE analysis of rigid PUR foam machining using the ALE method and to verify the results by comparison with the results of experiments. To assess the validity of the FE model, we conducted a more detailed analysis of its energy balance. The total energy $E_{T}$ can be set from the energy balance in the system as

(7) $$\begin{array}{c} E_{T}=E_{I}+E_{V}+E_{FD}+E_{KE}+E_{IHE}-E_{W}-E_{HF} \end{array}$$

where $E_{I}$ is the internal energy (total strain energy), $E_{V}$ is the viscous dissipated energy, $E_{FD}$ is the frictional dissipated energy, $E_{KE}$ is the kinetic energy, $E_{IHE}$ is the internal heat energy, $E_{W}$ is the work done by external forces, and $E_{HF}$ is the external heat energy through external fluxes (adapted from [22]). When the material is machined, the dominant component of the energy is converted into total strain energy $E_{I}$, and internal heat energy $E_{IHE}$. The graph in Figure 8a shows the magnitude of the total energy $E_{T}$, the total strain energy $E_{I}$ and the internal heat energy $E_{IHE}$. The graphs show that the total energy of the system or the energy balance according to Equation (8) is very small. The internal heat energy $E_{IHE}$ is greater than the total strain energy $E_{I}$. Total strain energy was set as

(8) $$\begin{array}{c} E_{I}=E_{E}+E_{P}+E_{A}+E_{DMD} \end{array}$$

where $E_{E}$ is the elastic strain energy, $E_{P}$ is the inelastic dissipated energy, $E_{A}$ is the artificial strain energy associated with constraints used to remove singular modes, and $E_{DMD}$ is the energy dissipated by damage (adapted from [22]). To verify that hourglassing was not a problem in the FE simulations, we evaluated the artificial strain energy $E_{A}$ more closely. For control of hourglass deformation, artificial strain energy $E_{A}$ by the accumulated artificial strain energy was used . Because energy is dissipated through plastic deformation, $E_{f}\gg E_{E}$ applies a comparison $E_{A}$ to an energy quantity that includes the dissipated energy as well, as the $E_{E}$ is most meaningful in this analysis (Figure 8b). $E_{A}$ is approximately 10.3% of $E_{f}$, therefore hourglassing does not influence results.

**Figure 9:**
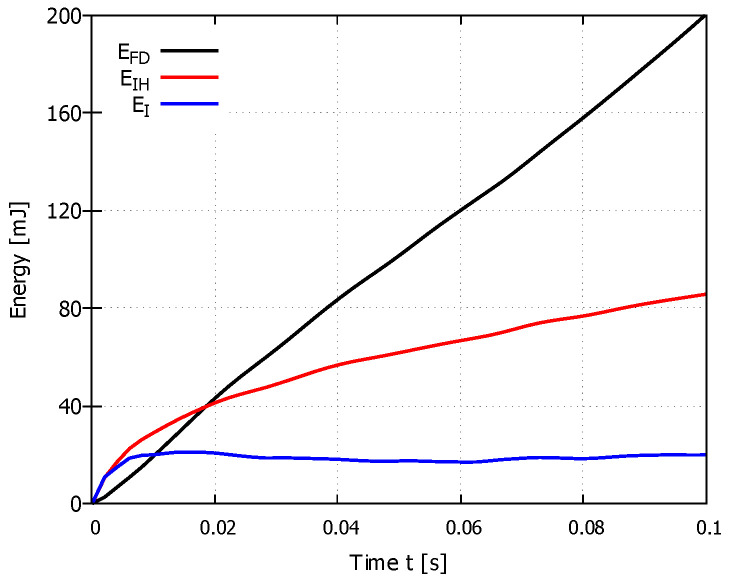
Graphs of frictional dissipated energy $E_{FD}$, internal heat energy $E_{IH}$, and total strain energy $E_{I}$ in the FE model during a cutting simulation with constant cutting speed of 500 mm/s and a sample density 40 PCF.

Figure 9 shows the magnitude of the total strain energy $E_{I}$, internal heat energy $E_{IH}$, and frictional dissipated energy $E_{FD}$. The graph for PUR foam with a density of 40 PCF and constant cutting speed of 500 mm/s shows that most energy is dissipated into friction and that substantially less energy is dissipated into material deformation. Frictional dissipated energy $E_{FD}$ was set as 31.9 mJ (10 PCF), 184.0 mJ (25 PCF), and 200.5 mJ (40 PCF) for a constant cutting speed of 500 m/s. From the results, $E_{FD}$ is only somewhat dependent on the sample density: the increase in $E_{FD}$ between densities of 10 and 25 PCF is 476.8%, whereas the increase in $E_{FD}$ between densities of 25 and 40 PCFs is only 8.9%. The magnitude of frictional dissipated energy $E_{FD}$ is substantially more dependent on the magnitude of the cutting speed. A frictional dissipated energy $E_{FD}$ of 55.9 mJ, 200.5 mJ, and 238.3 mJ was found at cutting speeds of 133, 500, and 833 mm/s for the 40 PCF sample, representing $E_{FD}$ increases of 258.7% at 500 mm/s and 118.7% at 833 mm/s, compared with the $E_{FD}$ at 133 mm/s.

Another parameter suitable for evaluating the FE model is the temperature at the point of contact of the tool with the material and its decrease with increasing distance from the tool on the material surface (Path A) and towards the inside of the material (Path B). The temperature graph in Figure 7 shows that the FE model works very well. The temperature value decreases substantially with increasing distance from the tool, consistent with the experimental results. The proposed FE model is sensitive to changes in material density and cutting speed. On the basis of these results, the results obtained with the ALE method match the experimental data well and can be used to accurately simulate the machining of rigid PUR foam.

Experimentally measured mechanical properties of rigid PUR foam were set as a function of the temperature when measurements were realised with specimens under both compression and tension. A total of seven samples were measured for each of the three PUR foam densities; this number appears to be statistically relevant because the measured data show a relatively low variance (13% or less).

The determination of the maximal temperature on the drill tip during drilling in PUR foams with different densities (10, 25 and 40 PCF) and various cutting speeds (800, 3000, and 5000 rpm) was performed experimentally.

For each combination of sample density and cutting speed, experimental measurements were performed on seven samples. This number appears to be statistically relevant because the measured data showed a relatively low variance (as high as 10%).

## 5. Conclusions

The main aim of this work was to carry out a numerical FE analysis of rigid PUR foam machining and to verify the results by comparison with the results of experiments. We created a complex FE model for simulating a brittle material machining, namely PUR foam, whose material properties are highly temperature-dependent. In the FE model, a unique complex constitutive material model was used in which the dependence of deformation rate, damage initiation, damage propagation, and plasticity on temperature was implemented. Such a complex material model used for deformation and heat propagation in a brittle material has not been presented anywhere yet. The Arbitrary Lagrangian–Eulerian method was used for FE machining simulations concerning minimal chip formation in brittle materials. In the realised FE simulations, the effect of various density and cutting speeds on the amount of heat generated during machining of the rigid PUR foam was evaluated.

Results obtained from FE analyses are in good agreement with the results of experimental measurements. The FE models with a constant cutting speed of 500 mm/s and various PUR foam densities led to slightly higher T${}_{max}$ values, where the differences were 13.1% (10 PCF), 7.0% (25 PCF), and 10.0% (40 PCF). The same situation is observed for the simulation results related to various cutting speeds at a constant PUR foam density of 40 PCF, where the differences were 25.3% (133 mm/s), 10.1% (500 mm/s), and 15.5% (833 mm/s). The results of FE analyses agreed well when the model sensitively responded to changes in the density of the PUR foam and the cutting speed. Upon closer evaluation of the FE model, we found that the internal heat energy was greater than the total strain energy, in agreement with the real situation. To verify that hourglassing was not a problem in the FE simulations, we evaluated the artificial strain energy; given that the artificial strain energy was 10.3% of the total internal energy, hourglassing was not a problem and the FE model was valid. According to the obtained results, the ALE method provides a good match with the experimental data and can be used to accurately simulate the machining of rigid PUR foams. The ALE method allows simulation of a long-term continuous machining process effectively without annoying restrictions such as computing cost and or excessive finite element distortions.

Experimentally measured mechanical properties of rigid PUR foam were set as a function of the temperature when measurements were realised with specimens under both compression and tension. An accurate description of the mechanical properties of PUR foam of different densities depending on temperature has not yet been published, and the relevance of the measured data for their use in the FE simulations is relatively high. From the results of the experimental measurements, it is clear that PUR foam is a specific material and cannot be modeled as a linear elastic material in numerical simulations.

The determination of the maximal temperature on the drill tip during drilling in PUR foams with different densities (10, 25, and 40 PCF) and various cutting speeds (800, 3000, and 5000 rpm) was performed experimentally. For each combination of sample density and cutting speed, experimental measurements were performed on seven samples. This number appears to be statistically relevant because the measured data showed a relatively low variance (as high as 10%). The results of the experimental measurements show that the maximum temperature on the drill bit increases with increasing density of the PUR foam and increasing cutting speed. These results are in accordance with the predictions and were used only to verify the results of the FE analyses.

## Figures and Tables

**Table 1:** Material parameters of all PUR foams dependent on temperature.

	Equivalent Plastic	Fracture Energy	Density	Specific Heat	Conductivity
	Strain ${\bar{\varepsilon }}_{D}^{\mathrm{pl}}$ [-]	$G_{f}$ [J/mm${}^{2}$]	ρ [g/cm${}^{3}$]	$c_{p}$ [J/(kg.K)]	k [J/(m.s.K)]
	40 PCF				
298.15 K	0.0400	40.0			
363.15 K	0.0290	29.0	0.64	809	0.11
428.15 K	0.2242	24.2			
	25 PCF				
298.15 K	0.0155	6.23			
363.15 K	0.0323	3.28	0.40	1 400	0.06
428.15 K	0.1494	2.60			
	10 PCF				
298.15 K	0.0315	0.866			
363.15 K	0.0421	0.245	0.16	1 477	0.037
428.15 K	0.2174	0.102			

**Table 2:** Johnson–Cook model parameters for rigid polyurethane foams with density of 10, 25 and 40 PCF.

Sample	A [MPa]	B [MPa]	n	C	${\dot{\varepsilon }}_{0}$ [1/s]]	m	$T_{m}$ [K]	$T_{0}$ [K]
10 PCF	1.4670	20.6235	0.831567	0.012	1	7.5052	675.0	273.15
25 PCF	4.3345	169.377	0.942053	0.012	1	11.623	675.0	273.15
40 PCF	11.9248	92.6822	0.686915	0.012	1	8.730	675.0	273.15

**Table 3:** Mechanical properties of PUR foams measured in experimental tests.

	25 ${}^{\circ }$C		90 ${}^{\circ }$C		155 ${}^{\circ }$C	
**Sample**	**Tensile**	**Compressive**	**Tensile**	**Compressive**	**Tensile**	**Compressive**
	**Modulus (MPa)**	**Modulus (MPa)**	**Modulus (MPa)**	**Modulus (MPa)**	**Modulus (MPa)**	**Modulus (MPa)**
PCF 10	86.25±13.29	49.71±9.94	53.07±5.84	26.38±4.75	3.01±0.57	3.96±0.52
PCF 25	309.06±32.54	262.89±55.21	280.32±40.05	203.83±20.38	8.48±1.10	6.52±0.59
PCF 40	543.95±54.40	452.02±94.92	507.19±85.08	378.16±41.31	71.29±11.41	54.92±9.89

**Table 4:** Experimentally measured maximum temperature T${}_{max}$ [${}^{\circ }$C] for three analysed revolution speeds and three PUR foam densities.

Rotation Speed [rpm]	800	3000	5000
**Axial Speed [mm/min]**	**10**	**30**	**45**
10 PCF	36.07±2.3	61.90±2.2	63.33±4.4
25 PCF	62.87±1.7	97.17±5.2	105.80±9.3
40 PCF	83.17±1.2	116.63±8.5	123.67±9.5

**Table 5:** Maximum temperature T${}_{max}$ [${}^{\circ }$C] obtained from FE analyses for three analysed PUR foams with different densities (with constant cutting speed 500 mm/s) and various cutting speeds (with constant density of 40 PCF).

500 mm/s	10 PCF	25 PCF	40 PCF
T${}_{max}$ [${}^{\circ }$C]	70.0	104.0	128.3
**40 PCF**	**133 mm/s**	**500 mm/s**	**833 mm/s**
T${}_{max}$ [${}^{\circ }$C]	104.2	128.3	142.9

## Data Availability

Not applicable.

## Nomenclature

PUR	Rigid polyurethane foam
FEM	Finite element
ALE	Arbitrary Lagrangian–Eulerian
UV	Ultraviolet
PCF	Pounds per Cubic Foot
CNC	Computer Numeric Control
${\bar{\varepsilon }}_{D}^{pl}$	Ductile equivalent plastic strain (-)
$G_{f}$	Fracture energy (${J/\mathrm{mm}}^{2}$)
ρ	Density (${g/\mathrm{cm}}^{3}$)
$c_{p}$	Specific heat (J/kg.K)
*K*	Conductivity (J/m.s.K)
IR	Infra red
$T_{max}$	Maximum temperature (K, ${}^{\circ }$C )
$v_{R}$	Rotation cutting speed (rpm)
$v_{a}$	Axial cutting speed (mm/min)
*t*	Chip thickness (mm)
*f*	Friction coefficient (-)
*k*	Conductance (W/m.K)
$\bar{\sigma }$	Static yield stress (MPa)
*A*	Yeld strength (MPa)
*B*	Hardening modulus (MPa)
*C*	Coefficient of strain rate sensitivity (-)
*n*	Hardening coefficient (-)
*m*	Thermal softening coefficient (-)
$T_{0}$	Transition temperature (K)
$T_{m}$	Melting temperature (K)
${\bar{\varepsilon }}^{pl}$	Mises equivalent plastic strain (-)
${\dot{\bar{\varepsilon }}}^{pl}$	Mises equivalent plastic strain rate
${\dot{\varepsilon }}_{0}$	Reference strain rate (1/s)
η	Stress triaxiality
*p*	Preasure stress (MPa)
*q*	Mises equivalent stress
$\omega_{D}$	State variable
${\bar{\varepsilon }}_{0}^{pl}$	Equivalent plastic strain (-)
${\bar{\varepsilon }}_{f}^{pl}$	Equivalent plastic strin at failure (-)
${\bar{u}}_{f}^{pl}$	Equivalent plastic displacement at failure (-)
$E_{T}$	Total energy (mJ)
$E_{I}$	Total strain energy (mJ)
$E_{V}$	Viscous dissipated energy (mJ)
$E_{FD}$	Frictional dissipated energy (mJ)
$E_{KE}$	Kinetic energy (mJ)
$E_{IHE}$	Internal heat energy (mJ)
$E_{W}$	Work done by external forces (mJ)
$E_{HF}$	External heat energy (mJ)
$E_{E}$	Elastic strain energy (mJ)
$E_{P}$	Inelastic dissipated energy (mJ)
$E_{A}$	Artificial strain energy (mJ)
$E_{DMD}$	Energy dissipated by damage (mJ)
